# Supplementary material for: The transcriptional regulator EarA and intergenic terminator sequences modulate archaellation in Pyrococcus furiosus
Source: Front Microbiol. 2023 Nov 9;14:1241399. doi: 10.3389/fmicb.2023.1241399 (PMC10665913; doi:10.3389/fmicb.2023.1241399)
Supplement: Supplementary file 3 [file Data_Sheet_1.PDF]

Supplementary Material

The transcriptional regulator EarA and intergenic terminator sequences modulate archaellation in *Pyrococcus furiosus*

Richard Stöckl[1†], Laura Nißl[1†], Robert Reichelt[1], Reinhard Rachel[2], Dina Grohmann[1]*, Felix Grünberger[1]*

[1] Institute of Microbiology and Archaea Centre, Faculty for Biology and Preclinical Medicine, University of Regensburg, Regensburg, Germany

[2] Centre for Electron Microscopy, Faculty for Biology and Preclinical Medicine, University of Regensburg, Regensburg, Germany

† These authors contributed equally to this work and share first authorship

*** Correspondence:**
Corresponding Author

dina.grohmann@ur.de, felix.gruenberger@ur.de

Keywords: Archaea, Archaellum, transcriptomics, EarA, *Thermococcales*, single-molecule sequencing, transcriptional regulator

1 Supplementary Figures and Tables

1.1 Supplementary Figures

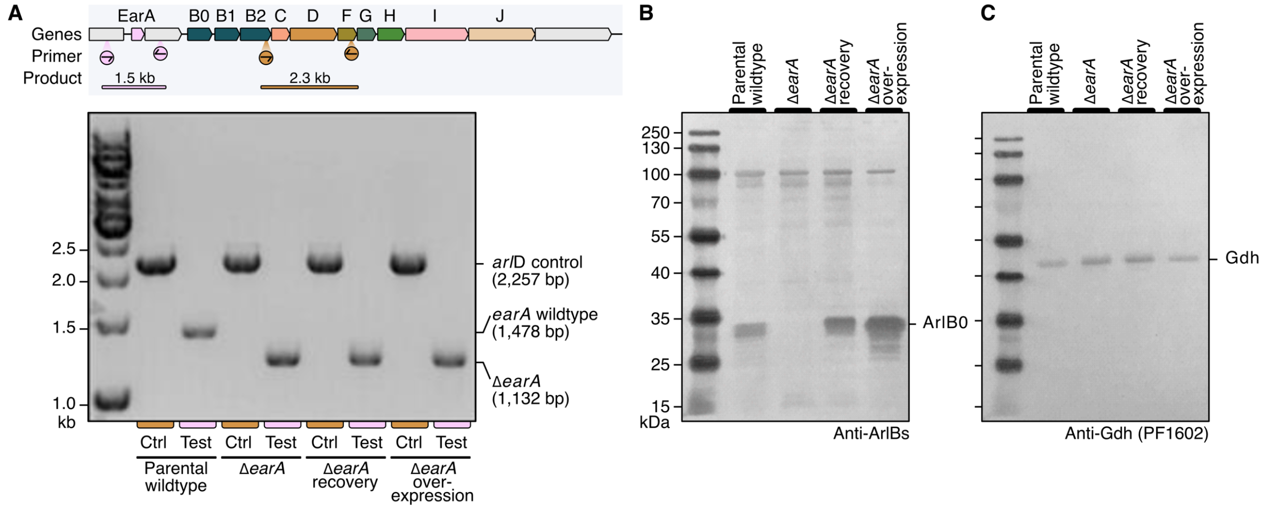

Supplementary Figure 1 | Confirmation of gene deletion and estimation of EarA expression levels in *P. furiosus* EarA deletion and recovery strains. A, Gene annotation of the *arl* gene cluster in *Pyrococcus furiosus*, with forward/reverse primers highlighted in circles (*arlD*: Fladup_100fw/Fladdo_100rw, *earA*: 0340do_113rw/0340up_100fw) and final PCR product in pink (*earA*) and orange (*arlD*). Results from PCR amplification of genomic DNA purified from the four strains used in this study using the two primer sets shown in panel A. **B**, Western Blot analysis of protein extract from the four strains used in this study using Anti-ArlB antibody and **C**, Anti-Gdh control.

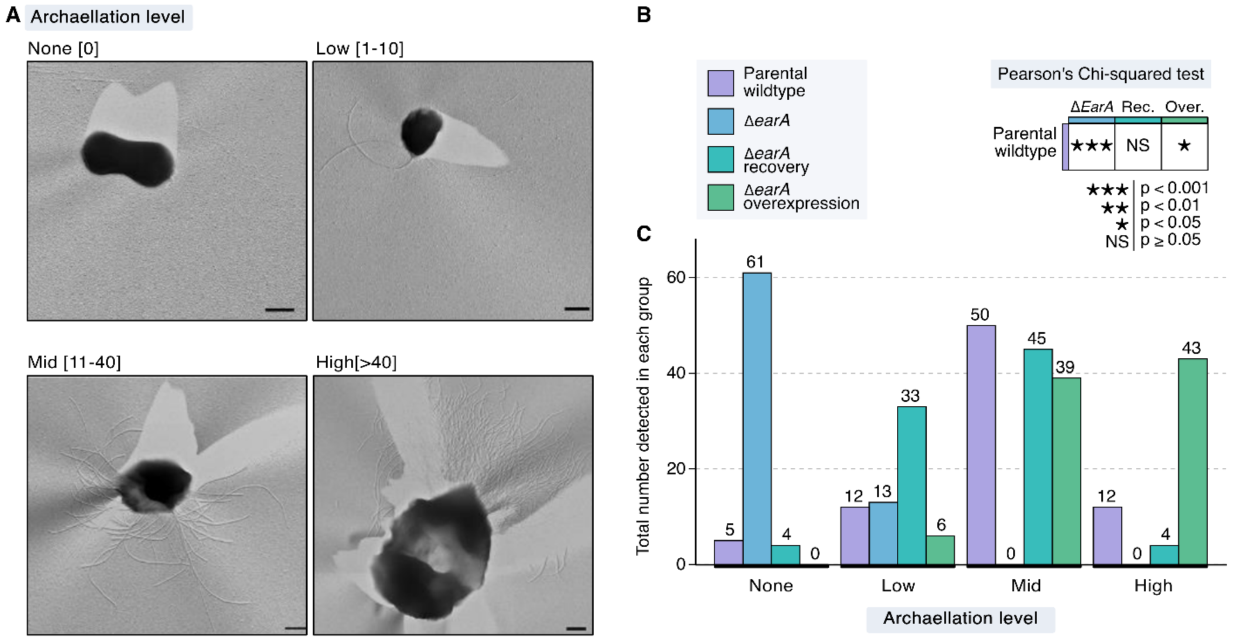

Supplementary Figure 2 | Systematic evaluation of archaellation in genetic *earA* variants. A, Representative TEM micrographs for the four levels of archaellation that were used to classify micrographs of the genetic variants, based on the number of visible archaealla as none (0), low (1-10), mid (11-40) and high (>40). **B**, Statistical test (Chi-squared test of mean number of observations in each level) to test significance of differential archaellation in the genetic variants. **C**, Total number of detected observations in each archaellation level color-coded by strain variant.

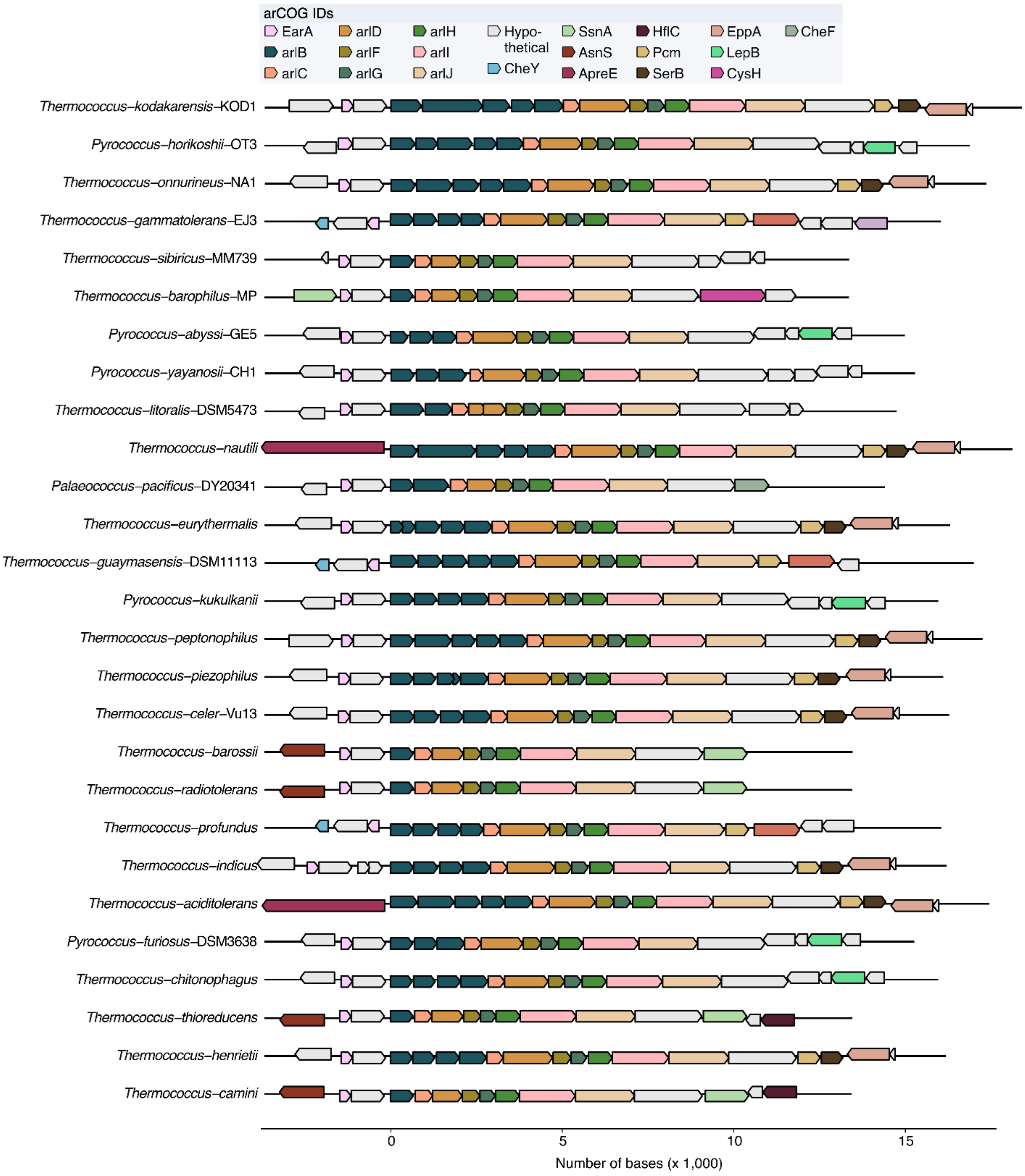

Supplementary Figure 3 | Genomic contexts of the *arl* gene clusters in *Thermococcales* species as defined by arCOG. HMM matches to protein sequences predicted by Prokka v. 1.14.6, centered on the *arlB0* gene (Seemann, 2014; Makarova et al., 2015). Strandness is indicated by the arrow directions. All gene clusters exhibit a conserved structure comprised of single copies of the genes *arlC-J* preceeded by one to five *arlB* paralogs. The gene coding for the transcriptional regulator EarA is located immediately upstream of the *arl* gene cluster.

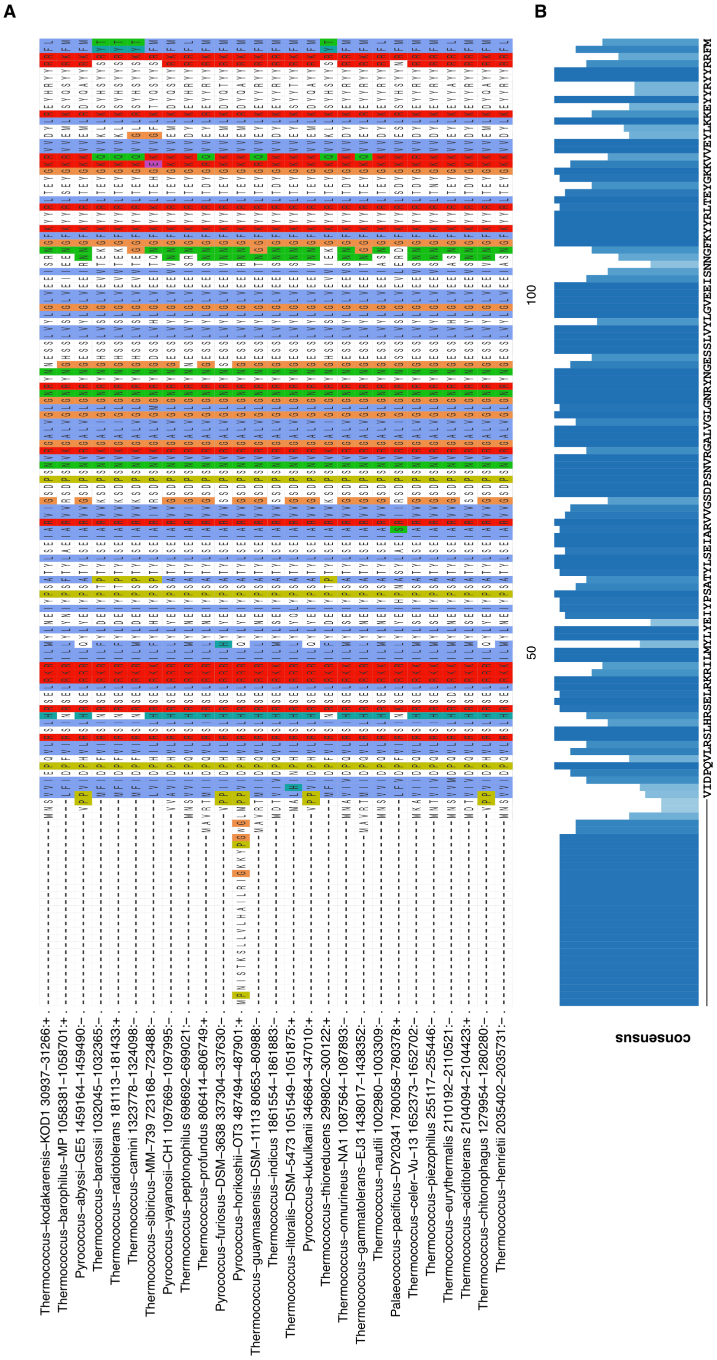

Supplementary Figure 4 | Multiple sequence alignment of EarA protein sequences in *Thermococcales*. **A**, Protein sequences predicted by Prokka v. 1.14.6 that matched to the EarA arCOG HMM were aligned using ClustalO (v. 1.2.3) (Seemann, 2014; Sievers and Higgins, 2014; Makarova et al., 2015). Residues are colored based on their properties and identities: hydrophobic (blue), positive charge (red), negative charge (magenta), polar (green), cysteines (pink), glycines (orange), prolines (yellow), aromatic (cyan). **B**,Sequence conservation is indicated by the bar char and the resulting consensus sequence is given below. Genomic position and strandness of the respective gene is indicated next to the organism name. All EarA sequences exhibit highly conserved regions intersperced with mostly conserved regions.

1.2 Supplementary Tables

Supplementary Table 1 | Used strains, plasmids, primer sequences, and antibodies

Supplementary Table 2 | Data summary of the downloaded reference genomes & genome annotation. (Summary as created automatically by the NCBI genomes page. For genome annotations, CDS are listed for each contig of the reference genomes with their genomic location as predicted by Prokka v. 1.14.6, with the respective arCOG annotation (Seemann, 2014; Makarova et al., 2015).

2 References

Makarova, K., Wolf, Y., and Koonin, E. (2015). Archaeal Clusters of Orthologous Genes (arCOGs): An Update and Application for Analysis of Shared Features between Thermococcales, Methanococcales, and Methanobacteriales. *Life* 5, 818–840. doi: 10.3390/life5010818.

Seemann, T. (2014). Prokka: rapid prokaryotic genome annotation. *Bioinformatics* 30, 2068–2069. doi: 10.1093/bioinformatics/btu153.

Sievers, F., and Higgins, D. G. (2014). Clustal Omega. *Curr. Protoc. Bioinforma.* 48. doi: 10.1002/0471250953.bi0313s48.
